# Impact of training and digital extension services on agricultural technology adoption and rice yields

**DOI:** 10.1371/journal.pone.0337456

**Published:** 2025-12-05

**Authors:** Apsara Karki Nepal, Dyutiman Choudhary, Naba Raj Pandit, Narayan Khanal

**Affiliations:** International Maize and Wheat Improvement Center (CIMMYT), Khumaltar, Kathmandu, Nepal; Rajiv Gandhi University, INDIA

## Abstract

Although rice is the primary staple crop in Nepal, its yields have not increased sufficiently to keep pace with population growth and food security needs. The key factors contributing to this challenge is inadequate agricultural extension services that meets farmers’ demand and the low adoption rate of technologies that could enhance rice productivity. This study evaluates the effects of sequential agricultural training and real-time digital extension services on the technology adoption and rice yield in Nepal’s Western Terai, where evidence on the effectiveness of such combined intervention remains limited. Using data from a stratified random sample of 1,396 households, including treatment, control-in, and control-out groups, we assess the impact of these interventions on technology adoption and rice yield. We apply quasi-experimental methods including treatment effects model (IPWRA), seemingly unrelated regression, and instrumental variable approaches. Our findings consistently indicate that smallholder farmers who receive agricultural training combined with digital extension services have 11.4–15.7 percentage points higher adoption rate of split urea application in three doses at the recommended stages of plant growth, resulting in a 35–56% increase in rice yield compared to the reference group. These results suggest that the combined effect of agricultural training and digital extension services significantly outperforms agricultural training alone in both technology adoption and yield improvement, which is one of the key contributions of this study. The findings highlight the potential of bundled approach to enhance rice productivity and strengthen food security. However, limited Internet access in farming communities and difficulties in interpreting digital advisories may constrain the broader adoption of these agricultural practices.

## 1. Introduction

In developing countries, a significant share of the population (40–50%) depends on agriculture for their livelihoods, with two-thirds engaged in small-scale (less than 2 ha), subsistence farming characterized by low productivity [1–3]. For these small-scale subsistence farmers, food insecurity remains a major challenge [4]. Farm mechanization combined with the adoption of new agricultural technologies– mainly modern agricultural inputs such as chemical fertilizers, improved seeds, and complementary micro nutrients which are included under agri-technology in this study – could enhance farm productivity. However, adoption is often constrained by fragmented land holdings [5], limited availability of complementary inputs [6,7], inadequate supply chain and market access [8], and weak agricultural extension services [9]. In these economies, population growth where land parcels are divided among family members as ancestral property, reduces landholding size, making the adoption of agricultural technologies even more challenging [1,10].

Agricultural extension services across South Asia, including Nepal, face persistent structural and operational challenges, limiting their ability to support smallholders in adopting productive, resilient, and efficient farming practices. Common constraints include underfunding of extension services [11], a shortage of skilled extension workers, weak research-extension-farmer linkages [12], limited use of digital technologies [13], and weak value-chain integration [14].

Rapid expansion of mobile technology offers an opportunity to bridge informational gaps in agricultural extension services [15]. With increased access to mobile phones, television, and internet services, agricultural extension can be provided digitally at minimal cost, as the marginal cost of operating such applications is virtually zero for information providers. Evidence suggests that digital extension services can improve input efficiency, enhance crop productivity, and reduce environmental impacts by providing tailored information that supports timely and effective farm level decision making [16–19]. Integrating digital services with in-person training and on-field demonstration further strengthens the adoption of modern agricultural technologies by addressing both knowledge gaps and skill limitations among smallholders.

The penetration of mobile phones in developing countries provides an effective medium for disseminating digital extension services to farming communities [20,21]. Such services not only help farmers make informed decisions but also act as nudges for taking necessary actions at the important dates including adherence to cropping calendars for fertilizer application, harvesting times, and weather forecasting, thereby reducing risks [19]. In addition, they provide farmers with access to price information, narrowing the profit margins of the agents who connect producers with consumers. Overall, leveraging digital technologies for agricultural extension has the potential to significantly increase the adoption of recommended agricultural practices and enhance crop yield [22].

Agricultural training and digital extension services could play a critical role in helping farmers make informed decisions regarding various aspects of rice crop cultivation, including seed choice, planting schedules, timing of fertilizer application, irrigation, pest and weed control. By providing timely guidance, these agricultural extension services help farmers optimize input use, particularly fertilizers, and thereby improve yields. However, the literature on the impact of agricultural training and digital extension services on farming decisions, technology adoption and yield is limited and mixed, as not all interventions are equally successful in producing the expected results [23].

The application of chemical fertilizer could go up or down depending on the existing situation. Fertilizer application, for instance, may increase or decrease depending on initial condition. For example, in Ghana, extension services raised application rates [24] while in China [25] and Bangladesh [26], they reduced overuse without reducing yields. A systematic review of 49 studies [27] highlights that the success of digital extension services depends on messages quality and relevance, user training, and integration with complementary interventions. Yet, digital extension often fails to improve input efficiency or yields due to low uptake or shallow engagement [28,29], mismatch with farmer needs [30], behavioral barriers, difficulties in translating messages into actions, message fatigue, measurement errors [31], the digital divide [32], and lack of complementary inputs or capital [22].

Though limited, some evidence from outside South Asia indicates that nutrient management is more effective when local training and demonstration are accompanied with digital reminders, which act as nudges that build farmer trust and encourage compliance with field-specific recommendations. However, these digital reminders are in terms of video demonstrations instead of real-time messaging [33]. Despite these insights, there is no Nepal specific literature that clearly distinguishes digital extension from traditional face-to-face training, and there is very little information on how extension services are delivered to the farmers, making it difficult to isolate their respective effects of digital extension vs. in-person training. Furthermore, adoption of agricultural technologies is shaped by socioeconomic characteristics of farmers, knowledge of agricultural practices, sociocultural beliefs, demographic characteristics, and institutional arrangements [34]. Since new and complex agricultural technologies often require precise knowledge and necessary skills [35], their adoption is constrained in contexts where traditional extension services are weak or absent [36]. Finally, the reliability of digital extension services is critical; without it, both adoption and benefits remains limited [37].

Rice is one of the main staple crops for more than half of the world’s population, with Asia producing about 90% of it [38]. To meet the rising demand driven by population growth, the existing literature suggests that rice production needs to be increased by over 26% by 2035, especially by smallholders [39]. In Nepal, rice accounts for 67% of total food consumption [40] and is cultivated on about 36% of the country’s arable land [41]. However, the cultivated land in Nepal has declined in recent years [42] for two main reasons. First, productive farm land is converted into settlement, where real estate profits exceed agricultural returns. Second, as labor shortage arise due to mass outmigration for temporary work, remittances are mainly used for consumption and urban land purchases, leaving marginal land fallow [43,44]. Consequently, cereal crop imports has been rising over time as Nepal struggles to meet its rice demand domestically [45].

**Fig 1:**
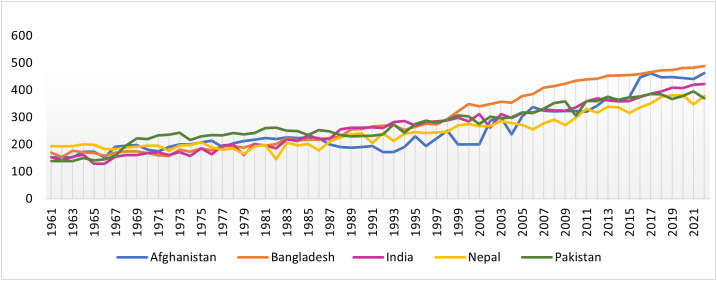
Rice yield trends in South Asian countries (1961–2022). Note. The relevant data is taken from FAOSTAT, October 7, 2024.

In addition, Nepal’s rice productivity remains low compared to other South Asian countries (Fig 1) due to limited access to improved agricultural technologies and exposure to extreme weather events such as floods and droughts [40]. Recent study reveal that despite technological progress, small and marginal farms in Nepal are less efficient, and women farmers face unequal access to technology, highlighting the need for greater technology adoption and equitable resource access [46].

In some areas where the climate is favorable, Nepal’s agricultural system is intensified, with two to three crops grown annually. This practice often leads to nutrient depletion due to imbalanced fertilizer use and ecological variations in cropping systems [47]. Since chemical fertilizers are not manufactured domestically, Nepal relies on imports. To maintain affordability, the government subsidizes imported fertilizers. However, limited budget allocation and inefficient institutional mechanisms for timely procurement and distribution have resulted in recurring fertilizer shortages during the rice cultivation season [46,48].

Under this backdrop, the main objective of this study is to examine the effectiveness of in-person agricultural training combined with field demonstrations, followed by a series of real-time digital messages related to the crop calendar for rice cultivation, inputs use, and associated farming activities among smallholder farmers. First, we consider the adoption of split application of urea (nitrogen fertilizer) at different stages of rice plant growth and other complementary inputs as a result of agricultural training and digital extension services. Second, we examine the impact of split application of urea (SAU) on rice yield. For this research, we use primary survey data collected using stratified random sample of 1396 farm households across six districts in the Western Terai of Nepal.

Controlling for a wide range of farm and farmer’s characteristics, we find that agricultural training and follow up digital extension services (e.g., the provision of texts and voice messages on the timing of inputs application) significantly promote the adoption of SAU at the recommended stages of rice plant growth, thereby increasing rice yield. We find that smallholder farmers who received both agricultural training and digital agricultural extension services exhibited 11.4–15.7 percentage points higher adoption rates of SAU at the recommended stages compared to farm households without such support (control group with mean adoption of 14%). Adoption of SAU at the recommended stages increased rice yield by 35–56% (equivalent to 40–70.5 kg/kattha or 1.2 to 2.1 metric ton/ha, where 1 ha = 29.5858 kattha, a local unit used in the study area), contributing to enhance food security. These results are robust across alternative model specifications and estimation methods. Since we use quasi-experimental approaches, including treatment effects models and instrumental variable methods (two-stage and three-stage least squares), these results have causal interpretation.

The major contribution of our research is threefold. First, it adds to the relatively limited literature in South Asia in general, and Nepal in particular, on the impact of the sequential agricultural training and field demonstration followed by real-time digital extension services, on technology adoption and rice yield. The majority of existing studies in South Asia examine either agricultural training [49] or the digital extension services [50,51], but not both, while in Nepal, there is very thin literature with clear specification of interventions types and delivery methods. Second, our results have causal interpretations, as we use multiple identification strategies to understand the impact of agricultural technologies on rice yield mediated by agricultural training and digital extension services. Third, this research is based on a carefully designed survey of smallholder farmers in Nepal, incorporating two types of comparison groups (control-in and control-out) to examine the spillover effects of training and field demonstration within villages. Finally, the findings provide evidence for scaling up the intervention applied in this study, at least across the Terai region and the broader Indo-Gangetic plain.

The remainder of the paper is organized as follows. Section 2 provides background information on the study areas, intervention strategies, and methodology. Section 3 describes the data, and presents the main results, along with additional evidence on heterogeneity and the mechanisms underlying our findings. Section 4 presents discussions, and section 5 concludes.

## 2. Materials and methods

### 2.1. Study setting and the intervention

This study examines two sequential interventions implemented by the International Maize and Wheat Improvement Center (CIMMYT) under the Maize Commercialization Model (MCM) in six districts of Nepal’s Western Terai, located within the USAID Feed the Future (FtF) zone of influence. Initiated in 2021, the MCM aims to promote sustainable and resilient production systems through public–private partnerships, emphasizing best management practices (BMPs), strengthened value chains, and improved market access for smallholder farmers.

The first intervention involved in-person agricultural training combined with field demonstration for rice farmers who are members of 41 local cooperatives, each comprising approximately 200–500 smallholder farmers. These cooperatives, engaged in fertilizer supply and crop marketing, collaborated with CIMMYT and local governments to facilitate the in-person training. Training sessions integrated theoretical and practical components, focusing on precision nutrient management using the 4R nutrient stewardship framework (right source, rate, place, and time for fertilizers application, including split use of urea in three doses), as well as improved seed use, irrigation efficiency, and pest and disease control. Gender equality and social inclusion (GESI) considerations were applied throughout, ensuring that over 40% of participating farmers were women or members of disadvantaged groups.

The second intervention introduced digital extension services in partnership with Geo-Krishi, a private AgriTech firm. A subset of farmers who had received agricultural training, also subscribed to the Geo-Krishi mobile application to receive real-time digital agro-advisories and weather-based messages. These advisories acted as behavioral nudges, reminding farmers to apply fertilizers and other inputs at optimal times.

### 2.2. Sampling and sample size

The survey used a multistage stratified random sampling technique to draw smallholder farmers from the defined study population. From the six study districts, five cooperatives were randomly selected per district, making a total of 30 cooperatives out of 41 in the study area, that have been collaborating with CIMMYT. It is important to note that not all villages within these districts are covered by cooperatives services. The selected cooperatives operate across different *Palikas* (the lowest administrative units or the local government is called *Palika*, which is either an urban or rural municipalities), and each *Palika* consists of a cluster of villages, where some villages are served by the cooperatives and others are not. Within villages where cooperatives provide extension services, not all farmers receive such services, as cooperative membership is voluntary. Therefore, our sample is subject to potential selection bias. This is not unique but rather a common challenge in development programs aiming to support targeted groups of beneficiaries, where interventions are implemented first, and researchers subsequently engaged to evaluate their impacts.

For power calculation, we use a baseline mean rice yield of 3.5 tons/ha and a standard deviation of 0.25 tons/ha, based on 10 years of historical rice yield data for Nepal (Fig 1). The power calculation suggests that with a minimum detectable effect of 5%, 30 clusters per arm (a total of 60 clusters), and 15 observations per cluster, statistical power remains very high (>95%) for intra-cluster correlation of 0.05. Even with a 10% attrition rate, the study remains adequately powered (>80%). Accordingly, we included 17 farm households per cluster in the control group. For the intervention group, we increased the sample size to 30 farm households per cluster (oversampling), as only a sub-set of households in these clusters received digital extensions services. This approach ensured sufficient representation of farmers who received both agricultural training and real-time digital extension interventions.

The research team obtained the list of all farmers served by the cooperatives (who received in-person training) in the selected villages, and randomly selected 30 farmers from the area covered by each cooperative within a *Palika*. From a district, we thus obtained 150 farm households (served by five cooperatives). In total, our sample includes 900 farmers across six districts, who received agricultural training (T1). Among these, some farm households who received T1, and also received digital extension services (T2).

The comparison group consists of two categories of farmers: control-in (T’) and control-out (T”). To understand potential spillover effects of the intervention (agricultural training and field demonstrations), we randomly selected five farm households from each area covered by the cooperative, but these farmers were not part of the intervention, making a total of 30 farmers per district who did not receive agricultural extension training but lived in the same villages as the intervention group. In our sample, we have 180 observations for control-in subsample. The control-out sub-sample was selected from villages outside the cooperatives’ operational areas. For this group, we identified village located near the cooperatives’ service areas (but not served by them) and randomly selected 12 farm households per village, making it 60 farm households per district and 360 farm households in total (T”). overall, our survey includes 1440 farm households that cultivated rice, maize or both crops in the previous season (before the survey).

Before conducting the household survey, verbal informed consent is received from each respondent (farmer). As the survey was conducted using electronic devices, each respondent’s informed consent is recorded electronically in each survey form (‘yes’ to proceed with the survey, and ‘no’ to drop the household in the absence of consent). If a respondent refused to participate in the survey, the enumerator moved to the next farmer on the randomized list. To accommodate refusals, the list contained more than 30 farmers per village, and once the quota of 30 farmers is fulfilled from the given village, the enumerator moved to the next sample village.

The research protocol received IRB approval from the CIMMYT Internal Research Ethics Committee on March 22, 2024, for one year (IRB #IREC.2024.008). Respondent recruitment and data collection took place between March 29, 2024 and ended on April 30, 2024.

The survey collected information from farm households for more than one linked study. In this study, we use a sub-sample of farm households that cultivated rice crops in the previous season. Therefore, the sample size is 1396 farm households (T1 – T2 = 428, T2 = 457, and T” + T’ = T3 = 511), after excluding farmers who cultivated only maize and those with incomplete information.

### 2.3. Methodology

#### 2.3.1. Basic framework.

**Fig 2:**
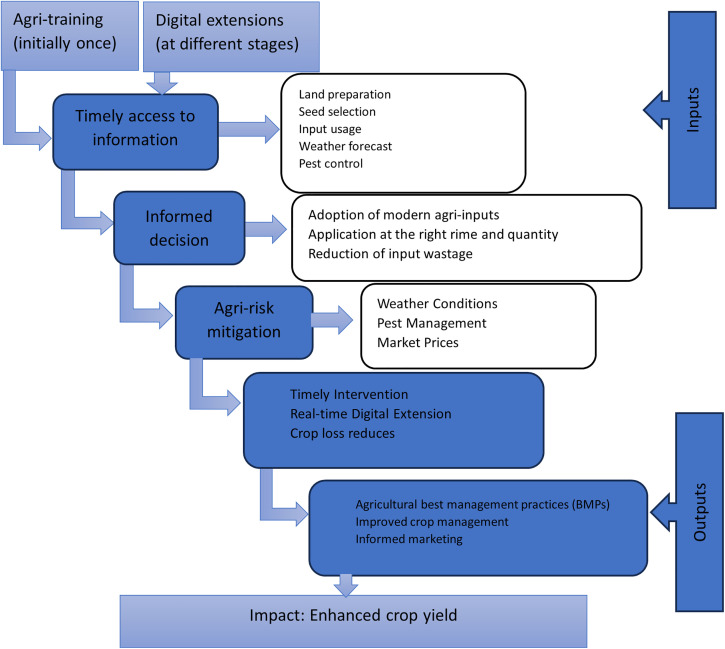
Impact pathway (Theory of change).

We develop a basic framework to illustrate the mechanism through which the interventions are expected to facilitate and encourage farmers to adopt specific agricultural practices for better outcomes. Fig 2 summarizes the impact pathways (theory of change), showing how the integration of agricultural training with real-time digital extension services is expected to contribute to optimizing input use, ultimately leading to higher yields.

#### 2.3.2. Empirical approach.

In this study, agricultural technology adoption refers to the use of split application of urea (SAU) (nitrogen) along with a set of complementary inputs recommended for rice cultivation, represented by a binary variable, Y. The recommended split application of urea divides the total nitrogen doses into three applications instead of a single dose at planting phase: the first dose is applied at planting, the second 21 days after establishment, and the third 50 days after establishment. In our sample, farmers either applied a single dose at planting rice (SAU0), or two doses (either at planting and 21 days (SAU1) or at 21 and 50 days (SAU2)) or all three recommended doses (SAU3). Accordingly, we modeled the split application of urea fertilizer as follows:


$$Y_{ij}=\;\beta_{\text{1}}.AgriTraining\;\left(T_{\text{1}i}\right)+\;\beta_{\text{2}}.AgriTraining\;\&\;DigitalExtension\;\left(T_{ti}\right)+X\beta +u_{ij}$$ (1)

where *Y*_*ij*_ is a binary (0/1) outcome variable of type *j* (split application of urea fertilizer represented by SAU1, SAU2 and SAU3, or other complementary inputs, such as rice sees, or other soil micronutrients) for household *i* and *X* is a vector of control variables, including farm and farmer’s characteristics; β’s are the regression coefficients to be estimated, and *u*_*ij*_ is a white noise error term, assumed to be normally distributed with zero mean and constant variance. The constant term is suppressed in Eq (1).

#### 2.3.3. Estimation strategy.

**2.3.3.1. Adoption of agricultural technologies:** For this study, we used cross-sectional data; therefore, estimating Eq (1) using the ordinary least squares (OLS) method may lead to selection bias, as the interventions were not randomly assigned. Therefore, we use a treatment effects methods, mainly, the inverse probability weighted regression adjustment (IPWRA) method, which is a quasi-experimental approach, for estimating causal impact of the intervention using observational data [52,53]. The method is briefly summarized below.

The IPWRA method is particularly suitable for our context, where program placement is non-random and simple comparisons of outcomes (e.g., split application of urea) between treated and control households could suffer from selection bias if the differences in pre-treatment characteristics are not adequately controlled for. For simplicity, we denote the outcome variable as Y, representing the adoption of a particular agricultural technology (fertilizer doses, type, and seed variety, etc.).

Let *Y*_*j*_*(1)* and *Y*_*j*_*(0)* denote the potential outcomes for household *i* in terms of the adoption of agricultural technology *j* under treatment (1) and control (0), respectively. The treatment status is indicated by T, where T_*i*_ = 1 if the household *i* received the treatment, and 0 otherwise. The observed outcome is therefore given by Eq (2):


$$Y_{i}=\;T_{i}\;.\;Y_{i}(\text{1})+\left(\text{1}-T_{i}\right)Y_{i}(\text{0}).\;$$ (2)

We are interested in estimating the average treatment effect on the treated (AETE), which is defined in Eq (3):


ATET=E[Y(1)−Y(0)|T=1]. (3)

Eq (3) measures the causal impact of the intervention on the technology adoption among households that received the intervention. The identification relies on the conditional independence assumption (CIA) – that is, conditional on a set of observable characteristics (X) of households and farms, the treatment assignment is independent of the potential outcomes. This assumption could be indirectly verified using covariate balance checks before and after matching, examining overlap in propensity scores, placebo tests, and sensitivity analysis. These diagnostic tests are presented in the results section.

The IPWRA estimator is often considered as doubly-robust [54] since it combines two strategies: a) inverse probability weighting (IPW), which uses estimated propensity scores to create a pseudo-population in which covariates are balanced across groups; and b) regression adjustment (RA), which models the outcome variable (split application of urea or other agricultural technologies) as a function of treatment status and covariates. Since our study involves three level of treatment, we use multinomial logit model to estimate the propensity score.

**2.3.3.2. Robustness:** The split applications of urea at different states of rice plant growth are inter-dependent and the decision needs to be made at the beginning of the crop establishment. Therefore, we use a seemingly unrelated regression (SUR) model, which accounts for three possible but interdependent decisions that farmers can make, represented by *Y*_*1*_, *Y*_*2*_ and *Y*_*3*_. In this context, *Y*_*1*_ denotes the decision to apply urea fertilizer during planting rice and 21 days after establishment (SAU1); *Y*_*2*_ represents the decision to apply urea fertilizer after 21 days and then 50 days of rice planting (SAU2); and *Y*_*3*_ refers the decision to follow all three recommended doses of split applications of urea into three stages applied during planting, 21 days after planting and 50 days after planting rice (SAU3). In our case, other complementary inputs include MOP (muriate of potash), boron-zinc, farmyard manure (FYM), and hybrid or open pollinated (OPV) seeds, which are typically decided before planting rice.

#### 2.3.4. Rice yield model.

**2.3.4.1. Two-stage and three-stage least squares approaches:** Rice yield can be modeled using the conventional production function approach, as shown in Eq (4):


$$Zi=f\left(Y|X\right)+v_{i}$$ (4)

Where, *Z*_*i*_ is the rice yield by household *i*, Y is a vector of agricultural technologies (inputs), *X* is a vector of farm and farmer’s characteristics, and *v*_*i*_ is an error term. In practice, input usage per unit of land, such as *c* kg of urea per kattha, is often highly correlated, as farmers tend to apply inputs in fixed proportion and fixed amounts per unit of land. In the absence of variation in input usage per unit of land, estimation of the production function becomes problematic due to high multicollinearity among inputs. Therefore, we opt for two-stage least squares (2SLS) methods, using agriculture-related training and digital agricultural extension services as instruments. These interventions do not directly influence rice yield (the outcome variable), they however are highly correlated with input choices, which in turn, affect rice yield. Agricultural training and real-time digital messages (as a nudge) enhance input efficiency by ensuring that inputs are applied at the right time and in the right amounts to increase rice yield. In this case, instead of Eq (4), we estimate the following Eq (5):


$$\begin{array}{c} Z\;=\;\;\alpha \;\hat{\;Y}+\;X\beta +\varepsilon \end{array}$$ (5)

where α measures the change in rice yield due to the adoption of agricultural technology among farmers who received training on agricultural practices and digital messages at different stages of rice plant growth, compared to those farmers who did not receive such training and digital messages, all else being equal.

**2.3.4.2. Quantile regression:** Quantile regression allows a more comprehensive modeling of the conditional distribution of the outcome variable (Z), rice yield in this case, especially in the presence of heteroskedasticity among observation, and is less influenced by extreme values or outliers in the dependent variable. This is because instead of modeling the conditional mean (as in OLS), quantile regression models conditional quantiles of the outcome, which makes it robust to nonconstant variance and heavy tails [55–57]. Instead of estimating a single mean, which is constant across the different values of the X (the covariates), quantile regression allows variation in the effect of Y, conditional on X, on the outcome variable, Z [58,59].

## 3. Results

### 3.1. Variables definition and summary statistics

As described earlier, our data source is a primary household survey of smallholder rice farmers distributed across six districts of western Terai in Nepal. Table 1 presents the descriptive statistics and definition of the variables used in the analysis.

**Table 1:** Variable definitions and summary statistics.

Variable		Treatment T1 (Ag. training only)	Treatment 2 (T2) (Ag. training & digital message)	Control (T3)
*Output variables*	Definition	Mean	Mean	Mean
Rice Yield	Kg/kattha^¥^	147.85	153.27	140.49
*Inputs usages*				
SAU1	1 if Urea is used at land preparation time and at 21 days of planting rice, else 0	0.20	0.19	0.24
SAU2	1 if Urea is used at 21 days and 50 days after planting rice, else 0	0.39	0.39	0.22
SAU3	1 if Urea is used at all 3 stages, else 0	0.23	0.26	0.14
MOP	1 if MOP use before planting rice, else 0	0.41	0.44	0.18
Hybrid seeds	1 if farmer used hybrid rice seeds, else 0	0.47	0.41	0.38
OPV seeds	1 if farmer used open pollinate rice seeds, else 0	0.61	0.74	0.69
	*Control variables*			
Extra Travel	1 if farmer is required to travel more than once to get fertilizer/season, else 0	0.66	0.60	0.62
Female	1 if respondent is woman, else 0	0.66	0.56	0.59
Age	Age of respondent (years)	43.86	43.14	44.3
Hill Dalit	1 if the respondent is disadvantaged social group of hill origin, else 0	0.04	0.03	0.08
Madeshi	1 if respondent’s ethnicity is Madesh origin, else 0	0.02	0.01	0.09
Hill Janajati	1 if respondent’s ethnicity is hill indigenous origin, else 0	0.1	0.12	0.08
Terai Janajati	1 if respondent’s ethnicity is Terai indigenous origin, else 0	0.45	0.43	0.46
Family size	Number of family members	5.35	5.21	5.63
Experience	Farming experience of respondent (years)	25.94	24.4	26.4
Ownership	1 if land is owned by women, else 0	0.18	0.14	0.16
Education	Respondent’s years of schooling	5.05	6.66	4.85
Land share	Share of land used for rice cultivation	1.86	1.86	2.18
Canal irrigation	1 if canal irrigation is available, else 0	0.2	0.27	0.26
Canal/Tubewell	1 if both canal and deep tubewell irrigation is available, else 0	0.17	0.17	0.12
Land parcels	Number of land parcels	2.79	2.76	2.57
Less fertile	1 if land is less fertile, else 0	0.61	0.48	0.67
Mini-tiller	1 if farmer uses mini-tiller, else 0	0.2	0.31	0.17
Thresher	1 if the farmer uses thresher, else 0	0.98	0.96	0.99
Fertilizer availability	1 if famer report enough fertilizer available, else 0	0.87	0.86	0.81
Cooperative	Distance between household & cooperative (km)	1.21	1.00	1.12
Market	Distance to input market (km)	4.34	3.94	4.02
Sample size	No of observations	428	457	511

Note. T1 - Ag. extension training only; T2 - Ag. extension training * digital messages; T3 = T’ + T”, sum of control-out and control-in. ^¥^Approximately 1 ha ≈ 29.5858 kattha.

Table 1 provides the means and standard deviations of the variables used in the analysis, divided into three subsamples: T1, T2 and T3. Urea usage seems to vary across these groups. A larger proportion of farmers in the control group used urea during land preparation and 21 days after rice planting (24%), whereas relatively fewer farmers who received agricultural training and digital extension services used urea during those two periods. However, approximately 26% of the sampled farmers in group T2 used urea in all three recommended stages of rice plant growth, compared to only 14% in the control group. The mean rice yield is 153.3 kg/kattha for farmers who received both treatments; and it is 140.5 kg/kattha for farmers in the control group.

The ownership of mobile phones is notably high among farming households. In our sample, approximately 88% of farmers had mobile phones, and about 34% reported receiving agricultural digital extension services. Furthermore, a significant majority (97%) of farmers who had not yet received the digital extension services expressed strong interest in receiving such services.

Table 1 indicates that most variables are balanced among the three subsamples: T1, T2 and T3, suggesting that the sampling method helped mitigate most of the sample selection biases. Demographic characteristics such as age and gender of respondents and household size are comparable across subsamples. Similarly, community characteristics, including distance to cooperatives, distance to markets, and the need to travel more than once to obtain the required amount of chemical fertilizer, are comparable among the subgroups of farm households. Approximately 60% of farm households in all subsamples reported having to travel more than once to obtain the required amount of fertilizer for rice cultivation in the last season (which refers to the rice season right before the survey). On average, respondents had over 25 years of farming experience and 5 years of schooling, and about 15% of households had land registered in the name of a woman family member. Farm characteristics are also comparable across the subsamples, where each group has approximately three land parcels on average, and since they are smallholders, they often share-in land for rice cultivation (thus the ratio of rice cultivated area to total land holdings is greater than one in all three subsamples). However, more farmers in the control group (T3) reported having less fertile land, and access to irrigation was also lower among them.

### 3.2. Impact of interventions on input choices

In this section, we discuss the results of the empirical analysis. We begin by examining the impact of agricultural training and digital extension services on the application of agricultural inputs (referred as agricultural technologies). Next, we present the result of the robustness checks. Finally, we analyze the effect of agricultural technology adoption, specifically the use of SAU as a key input on rice yield and discuss potential heterogeneities.

#### 3.2.1. Impact of the intervention on the adoption of agricultural technologies.

Table 2 summarize the results from the IPWRA estimator on the impact of the interventions on the split application of urea and the choice of rice seeds. In our sample, farmers applied urea to rice at different stages of plant growth; however, not all farmers followed the recommended three doses. The first three columns of Table 2 represent three different approaches to splitting urea into more than one dose. In column (1), the outcome variable is the application of two doses of urea, one at the time of planting rice and the second 21 days after rice planting. In Column (2), the outcome variable is also two doses of urea, where the first dose is applied 21 days after planting and the second dose 50 days after planting. In Column (3), the outcome variable is the recommended split application of urea (all three doses) at different stages of rice plant growth (at planting, at 21 days, and at 50 days after planting).

**Table 2:** Impact of intervention on input usages (urea and seeds).

IPWRA	SAU1	SAU2	SAU3	Hybrid rice seeds	OPV rice seeds
ATET					
T1 vs. T0	−0.012	0.160***	0.062**	0.072**	−0.078**
	(0.027)	(0.031)	(0.025)	(0.032)	(0.031)
T2 vs. T0	−0.032	0.159***	0.114***	0.02	0.046
	(0.027)	(0.031)	(0.027)	(0.033)	(0.031)
Mean of potential outcome	0.225***	0.233***	0.142***	0.393***	0.692***
	(0.019)	(0.020)	(0.016)	(0.022)	(0.021)

Note. IPWRA results where robust standard errors are in parentheses; * p < 0.10, ** p < 0.05, *** p < 0.01. SAU refers to the split application of urea, which has three different combinations: SAU1 refers to two doses applied at planting and 21 days after; SAU2 refers to two doses applied at 21 and 50 days after planting; and SAU3 refers to all three recommended doses.

Our results indicate that there were no significant differences in SAU at the time of land preparation and 21 days after planting between the farmers who received the treatments and those who did not (column 1). This is expected, as applying urea before rice planting is a common practice among farmers, and around a quarter of farmers applied urea at 21 days of rice planting. However, there were significant differences in SAU at 21 days and 50 days after rice planting (column 2), as well as in the use of urea at all three stages of plant growth (SAU3, column 3), compared with the comparison group of farmers. In relative terms, the probability of full compliance (SAU3) was much higher among farmers who received treatment T2 compared with those who received treatment T1 (11.4 vs. 6.2 percentage points against the control mean of 14%). The intervention (T1 or T2) improved SAU2 by approximately 16 percentage points (against of the sample mean of 23.3%).

We also examine the use of seed varieties under different treatments since these interventions provided farmers with information beyond fertilizer use, including seed choices. Results from Table 2 suggest that agricultural training helps farmers adopt hybrid seeds, mainly replacing the open pollinated variety (OPV) due to the perceived yield gain from the hybrid seeds. However, the impact of treatment T2 is not significantly different from zero for both hybrid and OPVs seeds. This may be explained by the fact that seed choice is typically made before farmers begin their cropping season, and the follow-up digital information provided later in the rice cultivation cycle may have little effect, since the decision on seed choice has to be made in the biggening of the cropping season.

#### 3.2.2. Complementary inputs.

We also consider how the interventions affect the use of micronutrients (complementary inputs), such as muriate of potash (MOP), boron-zinc, and farmyard manure. We did not consider DAP since there is no variation among the farmers in terms of DAP application. Table 3 shows the results (ATET) from the IPWRA estimator.

**Table 3:** Impact of interventions on the use of micronutrients and farmyard manure.

ATET (IPWRA estimator)	MOP	Boron & Zinc	Farmyard Manure
T1 vs. T0	0.194***	0.040***	0.206***
	(0.029)	(0.013)	(0.032)
T2 vs. T0	0.252***	0.074***	0.224***
	(0.031)	(0.016)	(0.033)
Potential outcome (mean of control group)	0.177***	0.018***	0.375***
	(0.017)	(0.006)	(0.022)

Note. Robust standard errors in parentheses; * p < 0.10, ** p < 0.05, *** p < 0.01.

The results indicate that the probability of using all three complementary inputs increases significantly, particularly when farmers receive treatment T2 (Table 3). For example, under treatment T2, the probability of using MOP increases by 25.2 percentage points, that of boron-zinc by 7.7 percentage points, and farmyard manure (FYM) by 22.4 percentage points. Under treatment T1, these impacts are somewhat smaller but remain significant (Table 3).

#### 3.2.3. Balancing test.

The IPWRA estimator provides a reliable estimate of the impact of the interventions (ATET) if the standardized (weighted) difference (SD) is below 0.10 and the variance ratio (VR) is between 0.5 and 2.0. Table 4 reports these statistics.

**Table 4:** Covariate balance (standardized differences and variance ratios with and without weighting).

	Standardized differences (SD)		Variance ratio (VR)	
Covariates (used for IPWRA)	Raw	Weighted	Raw	Weighted
**Case I: T2 vs. T0**				
Female	0.135	0.000	0.933	1.000
Age	−0.033	0.006	1.017	1.014
Farming experience	−0.054	−0.025	1.236	1.216
Years of schooling	0.039	0.010	0.986	0.966
Agricultural land area (kattha)	0.132	−0.032	1.620	0.704
Distance to input market	0.137	−0.018	1.340	1.132
**Case II: T2 vs. T0**				
Female	−0.073	0.005	1.022	0.997
Age	−0.097	0.005	0.911	0.901
Farming experience	−0.190	−0.038	0.934	0.908
Years of schooling	0.393	0.007	0.950	0.950
Agricultural land area (kattha)	0.214	−0.030	2.094	0.758
Distance to input market	−0.027	−0.047	0.942	0.834

Note. Standardized mean difference with and without weighing of the matching co-variates. For good matching, the rule of thumb is SD < 0.10; and 0.50 < VR < 2.0.

For the balancing test, we need pre-determined covariates, which are not affected by the intervention. Therefore, we use respondent’s gender, age, education, and farming experience which will not be affected by the intervention. Other variables that we consider are landholding size at the household level, which is also less likely to be changed in a single cropping season, as buying or selling land requires substantial investment; and the distance between input market and the location of farm. Both the parameters, the standardized mean differences (SD) and the variance ratio (VR) of these covariates are well within the acceptable range (below 0.047 for SD, and between 0.7 and 1.22 in case of VR). These statistics indicate that the covariates are well balanced under IPWRA, suggesting that results reported in Tables 2 and 3 are reliable, and can have a causal interpretation.

#### 3.2.4. Robustness check - seemingly unrelated regression (SUR).

To check the robustness of the results presented in Tables 2 and 3, we also analyze the data using alternative estimation methods. Our main analysis using IPWRA examines the effects of agricultural training and digital extension services on the split application of urea at different phases of rice plant growth, choice of rice seed, and the use of micro-nutrients. As indicated in the methods section, the decisions to apply majority of farm inputs including urea at different stages of rice plant growth are not independent of each other. Farmers and more likely to decide at the beginning of the planting season how they will use urea in rice cultivation (either as a single dose at the establishment phase or split into multiple doses). The choice of seed varieties is also interdependent. Therefore, we estimated three interdependent equations for SAU and two equations for seed varieties using two separate seemingly unrelated regressions (SURs). Table 5 reports these two sets of SUR results, where the first three columns present the SUR results for SAU fertilizer, and the last two columns report the results for rice seed choices.

**Table 5:** Results from the SUR models.

SUR model	2 doses urea (SAU1)	2 doses urea (SAU2)	3 doses of urea: (SAU3)	Hybrid rice seeds	OPV rice seeds
Intervention T1	0.015	0.175***	0.069**	0.090***	−0.079***
	(0.029)	(0.031)	(0.030)	(0.031)	(0.028)
Intervention T2	−0.078***	0.211***	0.157***	0.086***	−0.027
	(0.029)	(0.031)	(0.029)	(0.031)	(0.028)
R squared	0.139	0.211	0.104	0.298	0.347

Note. Clustered robust standard errors are in parentheses. Full results are provided in the supplementary information (S1 Table for SAU and seed choices); significance at the 10% level; ** significance at the 5% level; *** significance at the 1% level.

In our study, the interventions are agriculture training and digital extension services. All models include farmer characteristics such as gender, age, ethnicity, education, household size, farming experience, female land ownership, and farm characteristics as controls. We used district fixed effects to account for time-invariant unobserved heterogeneity at the district level. Standard errors are clustered at the village level, as the village is the final sampling unit from which our farm households were drawn. In the model, we consider both the total rice cultivated land area (in Kattha) and the share of cultivated land, where the results are consistent. The results presented in the tables include the share of rice cultivated land area. Since we have a long list of control variable, we report only the coefficients of the interventions in Table 5, while the full results are provided in supplementary information (SI, S1 Table).

Our results indicate that the impact of agricultural training with field demonstration (T1) is not significant for SAU1, while the farmers’ training and digital extension help farmers reduce the probability of using urea at establishment and 21 days after rice planting (SAU1) by 7.8 percentage points. This may be because farmers adjusted their urea application practices after receiving training and digital extension support. Applying urea during the establishment or transplanting phase in flood-irrigated rice fields often results in substantial nitrogen losses through volatilization, leaching, and denitrification [60,61]. On the other hand, the probability of applying urea in 21 and 50 days after rice plantation (SAU2) increased by 17.5 percentage points for those farmers who received agricultural training (T1) only and by 21.1 percentage points for those who received T2 (both interventions), compared to farmers who did not receive these interventions. The probability of using all three doses of urea (SAU3) is 15.7 percentage points higher for farmers who received T2 compared to the control mean, while it is only 6.9 percentage points higher for those who received intervention T1. Overall, intervention T2 has higher impacts compared to intervention T1 for SAU2 and SAU3. These results are consistent with those reported in Table 2.

It is interesting to observe the opposite impact of intervention T1 on the choice of seeds variety. Farmers who received the intervention T1 are 9 percentage points more likely to use hybrid seeds, while they are 7.9 percentage points less likely to use open pollinated varieties (OPV), compared to those using traditional variety. Since seed choice is made at the beginning of the rice season, intervention T2 is less effective, as the role of digital extension in the later phase would not influence seed selection. These results are again consistent with those reported in Table 2 (right panel).

### 3.3. Impact of input choices on rice yield

As noted in the methods section, agricultural training and digital extension services may not directly impact rice yield, but these informational inputs provide farmers with knowledge on agronomic practices including crops establishment timing; choice of seed types, fertilizer types, doses and timing; and application sequences, pesticide use, harvesting, and post-harvest management of the crop. By providing this knowledge, these interventions enable farmers to optimize the use of agricultural inputs for higher yields. Thus, we conceptualize the relationship between rice yield and interventions (agricultural training and digital extension services) as a two-step process. Input choices are crucial for rice yield, and can be influenced by the agricultural training and digital extension services that farmers receive. When farmers are well informed about the timing, quality and quantity of seed and fertilizers, as well as weather conditions and other relevant factors, they can make more efficient input decision, ultimately enhancing rice yields. Therefore, we use an instrumental variable (IV) approach (Eq (5)) to estimate the rice yield equation (Eq 4), where interventions T1 and T2 are used as instruments in the first stage. These interventions satisfy the fundamental criteria for valid instruments, they are not directly related to the outcome variable (rice yield) but are highly correlated with the endogenous variables (input choices), which in turn affect rice yield [62].

Table 6 shows the rice yield results, where we estimated three different models. As discussed above, since the SAU variables at different stages of rice plant growth are linked to each other, using all three options that farmers used for splitting urea application in the same rice yield equation results in multicollinearity. To address this issue, we estimated three separate models where the key input variables are a) three doses of urea as prescribed (SUA3), b) two doses of urea applied at 21 and 50 days after rice planting (SAU2), and c) two doses of urea applied during planting and at 21 days after planting (SAU1). In Table 6, we present two types of results from IV regression (columns 1 and 2) and three-stage least squares (3SLS) regression (column 3). Our IV results (column 1) indicate that the use of three doses of urea increases the rice yield by 70.5 kg per unit of land (kattha) where the average yield is 140 kg/kattha, compared to the reference group of households. The increase in rice yield is statistically insignificant when farmers use SAU2 after rice is planted. However, the use of SAU1 during planting rice and at 21 days after planting rice does not seem to have higher yield compared to the comparison group. This can be explained by the fact that urea application during the planting phase leads to substantial nutrient losses when rice is transplanted in flooded field, and hence low or no impact on yield.

**Table 6:** Impact of agricultural extension services on rice yield.

Dependent variable: rice yield or ln(rice yield)	IV Results	IV Results	3SLS Results	IV
Variables of interest: SAU	Rice yield (kg)	ln (rice yield)	ln (rice yield)	F-state
A: Three doses if urea: all three recommended doses	70.46***	0.46**	0.46**	12.82
	(23.70)	(0.22)	(0.20)	
B: Two doses of urea: 21 & 50 days	40.75***	0.30**	0.30**	25.29
	(14.17)	(0.13)	(0.13)	
C: Two doses of urea: land prep & 21 days	−91.23***	−0.53*	−0.53**	8.86
	(32.05)	(0.30)	(0.26)	

Note: In this table, we report the coefficient of the key variable of interest, and the full results are reported in the supplementary information, S2–S4 Tables. The interpretation of the coefficient, where the dependent variable is ln(rice yield) is slightly different: the coefficient needs to be transformed as (exp(*b*)-1)*100% to convert the coefficient into a percentage change (where *b* refers to the estimated coefficient). The first stage F-Stats are > 10 for case A and B; while it is < 10 for case C, a sing of weak instrument for case C, and hence we do not interpret these results.

Using rice yield (kg) directly as the outcome variable may create scale issues (overall sample mean is 145 with standard deviation 41 kg/Kattha). Therefore, in columns (2) and (3), we report results where the dependent variable is the natural log of rice yield (ln(rice yield)), which help address this scale issue. For the IV estimates, the first stage F-states are greater than 10, suggesting strong instruments. The Cragg-Doland under-identification test rejects the null of under-identification, with a chi-square value of 45.72, which is highly significant.

In this case, our results indicate that the average rice yield increases by approximately 56% when urea is applied at all three recommended stages of rice growth (row A in Table 6). When urea is applied in two split doses (at 21 and 50 days after rice planting), the yield increases by 35% (40.6 kg/kattha, row B). However, the first stage F-stat is less than 10, for row c, indicating weak instruments, and we do not interpret those results further due to weak instrument.

For the sensitivity analysis, we dropped the top 1% observations for the outcome variable (rice yield). These excluded values are more than three standard deviations above the mean. With this adjustment, the estimated impacts are slightly lower, 65 kg/kattha in the linear model, or 54% higher than the reference case in both IV and 3SLS regressions for all three doses of urea applications. Since these values are comparable to those reported in Table 6, a new table is not included in the text.

### 3.4. Understanding heterogeneity

There is some heterogeneity in the results presented thus far. As expected, the use of hybrid seeds, canal irrigation and farmer education have a positive impact on rice yield, whereas female land ownership, less fertile land, and larger land size have negative impact. The negative association between female land ownership and rice yield is noteworthy, possibly due to constraints faced by female land owners in cultivating rice, such as need for frequent travel to inputs market to obtain necessary inputs, including fertilizers.

**Fig 3:**
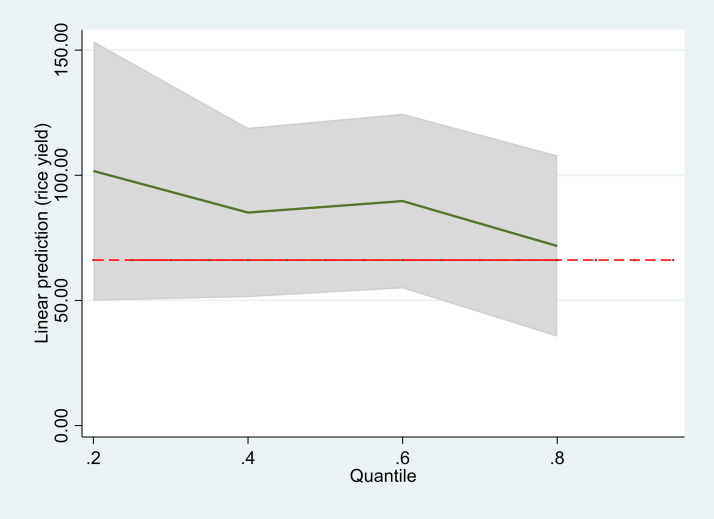
Heterogeneous effects of different covariates on rice yield (quantile regression). Note: The slope of the graph reflects the degree of heterogeneity. The flatter the line, the lower the heterogeneity.

To further explore the heterogeneous effect of the control variables, we conducted a quantile regression. Fig 3 displays the coefficients of the ‘three doses of urea use’ variable from the quantile regression. The estimates suggest that, the effect of applying three doses of urea on rice yield is considerably higher for the low-yield farmers (the vertical height of the solid downward slopping line gives rice yield by quantile) located at the lower end of the yield distribution (near the origin in the graph) compared to high-yield farmers in the upper quantile. This result suggests that the intervention is more effective in increasing rice yield among low-yield farmers, which could contribute to improved food security of these farm households.

## 4. Discussion

Rice is one of the main staple food crops grown in Nepal. However, rice yield has low in Nepal in comparison to other South Asian countries (Fig 1), and the area cultivated has been declining in the recent years. This phenomenon poses the question on food security in the country. To increase rice yield, CIMMYT has been providing agricultural training with field demonstrations. In recent years, this agricultural training has been supplemented by real-time digital extension services which deliver crop management information to farmers as timely nudges for enabling them to follow best management practices. These interventions provide farmers with skills and information, including reminders designed to prompt the timely applications of agricultural practices, such as the split urea application, seed selection, and the use of complementary micronutrients according to crop requirements, which in turn help increase rice yield.

### 4.1. Comparing key results with relevant literature

In our study, the combined effect of agricultural training and digital extension services increases the adoption of split use of urea application (two and three doses), resulting in improved rice yields of 35–56% (40–70.5 kg/kattha or 1.2 to 2.1 ton/ha). Direct comparison of these results with other studies is not feasible since other studies have either different types of intervention, crops, or geographical locations. An experimental study in Ethiopia [63] on cassava cultivation reports a 20–49% increase in cassava yield due to in-person training and video demonstration. A review study in Sub-Saharan Africa [64] finds that rice and maize yields increase by 36% and 65%, respectively, due to location specific input recommendations compared to a blanket recommendation, suggesting that our results are broadly comparable.

### 4.2. Digital reminders act as nudge

The key objective of the interventions is to encourage farmers to split urea into three equal doses, at the establishment phase, 21 days, and 50 days after planting, instead of applying it all at once during rice planting. This is particularly important since farmers tend to apply urea before rice planting, which results in substantial nutrient loss when flood irrigation technique is used (the dominant irrigation method for rice in Nepal). Our results suggest that farmers who receive T2 (in-person training + digital extensions) are more likely to adopt the recommended urea split, with an 11.4 percentage point increase compared to reference group (sample mean is 14%), whereas farmers who receive T1 (in-person training only) are only 6.2 percentage points more likely to adopt the recommended split. This indicates that digital extension services act as effective nudges for farmers to follow the recommended split urea application schedule. Our findings corroborate with the results reported in [65], which used an experimental method for understanding the impact of decentralized in-person intervention combined with digital (video) intervention. However, the quality and reliability of the information matters for the adoption of the recommended practices [66].

### 4.3. Issues of self-reported bias and generalizability

This study uses self-reported cross-sectional data, as the study is designed after the interventions. This approach is not uncommon in real-world development programs, where the main goal is to support marginalized and disadvantaged households and communities rather than solely for doing research and generate evidences. In the sample, rice yield is reported to be 140 kg/kattha (control group), which is closer to the value reported by FAO STAT (Fig 1) for 2022. Since FAO STAT reports an average yield of approximately 130 kg/kattha for the entire country, rice yield in the hills of Nepal tend to be lower than in the terai region. On this backdrop, the reported yield is not far from FAO reported statistics. Regarding generalizability, the study results may not be extended to the entire country, as the sample area is not representative of the whole country or even the terai region. However, given the proximity of the reported yield for terai region average, these results could be generalizable to the terai region of Nepal.

### 4.4. Removing institutional, policy, and market constraints is the key for wider adoption

Out study suggests that success of digital extension services has its limitations. In our sample, about 48% of the farmers who subscribed to digital extension services reported that mobile data packages or Internet services were expensive (unaffordable), and a similar percentage of farmers (48%) indicated that they either did not understand the digital messages or found them difficult to follow. Among the farmers who subscribed digital extension services, over 26% indicated that they were not comfortable using digital extension services, 17% stated that they never used the services, 78% used occasionally, and only 4% reported always using digital extension services for making agronomical decisions. These findings signify a large gap between the supply of the digital extension services and their use in agricultural decision making. Evidence from an experimental study [66] suggests that when information delivered through extension services is constrained due to lack of policy or institutional support for adopting the intended technology, the actual outcomes may be undesirable or unexpected. Therefore, alongside agricultural training and digital extension services, addressing other issues such as ensuring the timely availability of complementary inputs and removing supply constraints, is necessary for the wider adoption of agricultural technologies.

## 5. Conclusion

In this research, we evaluated the impact of agricultural training combined with real-time digital extension services on the split use of nitrogen fertilizer (urea), the use of complementary inputs, and seed choice (jointly called agricultural technologies). Furthermore, we examine how the adoption of these agricultural technologies, mediated by the interventions, affect rice yield.

Using a stratified random sample of 1396 farm households from six districts in the southwestern Terai of Nepal, we find that households in the intervention group (T2) are more likely, 11.4–15.7 percentage points higher than the control mean of 14%, to adopt three doses of urea at recommended stages of plant growth, resulting in higher rice yields of 35–56% (40–70.5 kg/kattha) compared to households in the comparison group. The probability of using two doses of urea (at 21 and 50 days after rice planting) also increases (16 percentage points above the control mean of 22%), but the impact on rice yield is lower (35%) than in the case of three doses of urea application (56%). These results suggest that rice yield is maximized when farmers apply three doses of urea at recommended stages of rice plant growth, facilitated by agricultural training combined with real-time digital extension services (T2). Agricultural training alone also boosts rice yield but less effective than when combined with digital extensions services.

However, given that farmers need to spend a significant amount of their income on mobile data or Internet services, limited access to digital extension services could create a digital divide, excluding poorer farmers even though the marginal cost of providing digital extension services to an additional farmer is negligible.

Expanding the digital network and reducing the costs of mobile data and Internet services for the farming community, through subsidies, for example, are potential strategies the government should consider to encourage adoption of digital extension services. Additionally, digital messages should be easy enough to understand, crop and location specific, reliable, and timely, enabling farmers to trust and adopt them for best management practices, ultimately improving rice yield.

Given the nature of this study as a real-world development program, our research relies on a single round of observational data. Future research should consider gathering longitudinal data through a randomized control trial (RCT) representing at least the entire terai region, the main rice producing area of Nepal. Another extension could involve conducting a cost-benefit analysis of different interventions (traditional vs. digital and combined) to help relevant stakeholders, local government, cooperatives, private firms and farmers, Identify the most efficient extension strategies.

## Supporting information

S1 Table Impact of Intervention on use of agricultural technology in different stages (SUR). (DOCX)

S2 Table Impact of agricultural technology adoption on rice yield (endogenous variable for the first stage: three doses of urea use). (DOCX)

S3 Table Impact of agricultural technology adoption on rice yield (endogenous variable for the first stage: 2 doses of urea: 21 & 50 days). (DOCX)

S4 Table Impact of agricultural technology adoption on rice yield (endogenous variable for the first stage: 2 doses of urea: land prep & 21 days). (DOCX)
