# Supplementary material for: Impact of training and digital extension services on agricultural technology adoption and rice yields
Source: PLoS One. 2025 Dec 5;20(12):e0337456. doi: 10.1371/journal.pone.0337456 (PMC12680215; doi:10.1371/journal.pone.0337456)
Supplement: S1 Table — (DOCX) [file pone.0337456.s001.docx]

Impact of Training and Digital Extension Services on Agricultural Technology Adoption and Rice Yields

**Supplementary Information (SI)**

We provide all the regression results in the supplementary information, where the key results are included in the main text.

S1 Table. Impact of Intervention on use of agricultural technology in different stages (SUR)

| SUR | (1) | (2) | (3) | (1) | (2) |  |  |
| --- | --- | --- | --- | --- | --- | --- | --- |
|  | Urea land prep 21days | Urea21 50days | Urea_  all stages | Seed variety | |  |  |
| VARIABLES |  |  |  | Hybrid rice | OPV rice |  |  |
|  |  |  |  |  |  |  |  |
| Agricultural extension training | 0.015 | 0.175*** | 0.069** | 0.090*** | -0.079*** |  |  |
|  | (0.029) | (0.031) | (0.030) | (0.031) | (0.028) |  |  |
| Access to digital agri-extension services | -0.078*** | 0.211*** | 0.157*** | 0.086*** | -0.027 |  |  |
|  | (0.029) | (0.031) | (0.029) | (0.031) | (0.028) |  |  |
| Control-in subsample | 0.058 | 0.108*** | 0.030 | 0.041 | -0.079** |  |  |
|  | (0.037) | (0.040) | (0.037) | (0.040) | (0.036) |  |  |
| Travel to get fertilizer more than once | -0.003 | 0.091*** | -0.068*** | -0.022 | 0.083*** |  |  |
|  | (0.025) | (0.027) | (0.025) | (0.027) | (0.025) |  |  |
| Female respondent | -0.041 | -0.000 | -0.045* | -0.018 | 0.013 |  |  |
|  | (0.026) | (0.029) | (0.027) | (0.029) | (0.026) |  |  |
| Respondent's age | -0.001 | -0.001 | -0.002 | -0.003** | 0.003* |  |  |
|  | (0.001) | (0.001) | (0.001) | (0.001) | (0.001) |  |  |
| Hill dalit | -0.028 | -0.118** | 0.087* | 0.012 | 0.035 |  |  |
|  | (0.049) | (0.054) | (0.049) | (0.053) | (0.048) |  |  |
| Madheshi | 0.005 | 0.025 | 0.020 | 0.082 | -0.022 |  |  |
|  | (0.058) | (0.063) | (0.059) | (0.063) | (0.057) |  |  |
| Hill Janajati | 0.082** | -0.105*** | 0.005 | 0.030 | -0.049 |  |  |
|  | (0.037) | (0.041) | (0.038) | (0.040) | (0.037) |  |  |
| Terai Janajati | 0.055** | -0.100*** | 0.079*** | 0.130*** | -0.026 |  |  |
|  | (0.027) | (0.030) | (0.029) | (0.030) | (0.027) |  |  |
| Number of household members | -0.003 | 0.000 | 0.000 | 0.000 | 0.003 |  |  |
|  | (0.002) | (0.002) | (0.002) | (0.002) | (0.002) |  |  |
| Farm experience (years) | -0.001 | -0.001 | -0.002* | 0.002 | -0.001 |  |  |
|  | (0.001) | (0.001) | (0.001) | (0.001) | (0.001) |  |  |
| Female land ownership | -0.018 | 0.028 | 0.039 | 0.023 | -0.051* |  |  |
|  | (0.029) | (0.031) | (0.029) | (0.031) | (0.028) |  |  |
| Respondent's years of schooling | -0.001 | 0.003 | -0.004 | 0.003 | 0.003 |  |  |
|  | (0.003) | (0.003) | (0.003) | (0.003) | (0.003) |  |  |
| Share of land for rice cultivation | -0.002 | 0.001 | -0.002 | -0.000 | 0.005 |  |  |
|  | (0.003) | (0.003) | (0.003) | (0.003) | (0.003) |  |  |
| Canal irrigation | 0.098*** | -0.027 | -0.041 | -0.154*** | 0.146*** |  |  |
|  | (0.028) | (0.030) | (0.030) | (0.030) | (0.027) |  |  |
| Canal and deep tubewell irrigation | 0.115*** | -0.093*** | 0.003 | -0.150*** | 0.183*** |  |  |
|  | (0.033) | (0.036) | (0.035) | (0.036) | (0.032) |  |  |
| Number of land parcels | 0.009* | -0.008 | 0.006 | 0.013** | -0.001 |  |  |
|  | (0.005) | (0.006) | (0.005) | (0.006) | (0.005) |  |  |
| Less fertile land | 0.031 | -0.059** | -0.004 | 0.006 | 0.006 |  |  |
|  | (0.022) | (0.024) | (0.022) | (0.024) | (0.022) |  |  |
| Use of mini-tiller | 0.042 | -0.054* | 0.007 | 0.039 | -0.011 |  |  |
|  | (0.028) | (0.030) | (0.028) | (0.030) | (0.027) |  |  |
| Use of thresher | -0.072 | 0.007 | 0.072 | 0.163** | -0.111 |  |  |
|  | (0.073) | (0.079) | (0.073) | (0.079) | (0.072) |  |  |
| Enough fertilizer available | -0.082*** | 0.132*** | 0.019 | -0.052 | 0.017 |  |  |
|  | (0.031) | (0.034) | (0.032) | (0.034) | (0.031) |  |  |
| Distance between household & cooperative | -0.012 | -0.031 | 0.016 | -0.044** | 0.023 |  |  |
|  | (0.017) | (0.019) | (0.018) | (0.019) | (0.017) |  |  |
| Constant | 0.324*** | 0.162 | -0.072 | 0.512*** | 0.428*** |  |  |
|  | (0.119) | (0.130) | (0.130) | (0.129) | (0.117) |  |  |
|  |  |  |  |  |  |  |  |
| Observations | 1,396 | 1,396 | 1,396 | 1,396 | 1,396 |  |  |
| R-squared | 0.139 | 0.211 | 0.104 | 0.298 | 0.347 |  |  |

Robust standard errors in parentheses *** p<0.01, ** p<0.05, * p<0.1
