# Supplementary material for: Impact of training and digital extension services on agricultural technology adoption and rice yields
Source: PLoS One. 2025 Dec 5;20(12):e0337456. doi: 10.1371/journal.pone.0337456 (PMC12680215; doi:10.1371/journal.pone.0337456)
Supplement: S2 Table — (DOCX) [file pone.0337456.s002.docx]

S2 Table. Impact of agricultural technology adoption on rice yield (endogenous variable for the first stage: three doses of urea use)

|  | (2) | (3) | (4) |
| --- | --- | --- | --- |
|  | IVreg2 | IVreg2 | IVreg3 |
| VARIABLES | Total rice production(kg) | ln_rice_yield | ln_rice_yield |
|  |  |  |  |
| 3 doses of urea: all three stages | 70.46*** | 0.46** | 0.46** |
|  | (23.70) | (0.22) | (0.20) |
| Female respondent | -1.33 | -0.02 | -0.02 |
|  | (3.46) | (0.04) | (0.03) |
| Respondent's age | 0.14 | -0.00 | -0.00 |
|  | (0.17) | (0.00) | (0.00) |
| Hill dalit | -11.17* | -0.09* | -0.09* |
|  | (6.69) | (0.05) | (0.05) |
| Madheshi | 1.65 | 0.01 | 0.01 |
|  | (6.30) | (0.05) | (0.06) |
| Hill Janajati | -3.28 | -0.09 | -0.09** |
|  | (4.91) | (0.06) | (0.04) |
| Terai Janajati | -12.78*** | -0.10*** | -0.10*** |
|  | (3.58) | (0.03) | (0.03) |
| Number of household members | -0.38* | -0.00* | -0.00 |
|  | (0.20) | (0.00) | (0.00) |
| Farm experience (years) | -0.01 | 0.00 | 0.00 |
|  | (0.15) | (0.00) | (0.00) |
| Female land ownership | -3.08 | -0.05 | -0.05* |
|  | (3.78) | (0.04) | (0.03) |
| Respondent's years of schooling | 0.57 | 0.00* | 0.00 |
|  | (0.37) | (0.00) | (0.00) |
| Share of land for rice cultivation | -0.70** | -0.01** | -0.01** |
|  | (0.30) | (0.00) | (0.00) |
| Canal irrigation | 4.54 | 0.04 | 0.04 |
|  | (3.48) | (0.03) | (0.03) |
| Canal and deep tubewell irrigation | 4.60 | 0.05* | 0.05 |
|  | (4.08) | (0.03) | (0.03) |
| Number of land parcels | -1.32** | -0.01* | -0.01 |
|  | (0.59) | (0.00) | (0.01) |
| Less fertile land | -10.01*** | -0.06** | -0.06*** |
|  | (2.73) | (0.02) | (0.02) |
| Use of mini-tiller | -0.49 | -0.00 | -0.00 |
|  | (3.36) | (0.03) | (0.03) |
| Use of thresher | -11.79 | -0.10* | -0.10 |
|  | (8.82) | (0.06) | (0.08) |
| Enough fertilizer available | -1.03 | 0.00 | 0.00 |
|  | (3.86) | (0.03) | (0.04) |
| Distance between household & cooperative | 0.54 | 0.01 | 0.01 |
|  | (2.05) | (0.02) | (0.02) |
| Distant to input market | 0.01 | 0.00 | 0.00 |
|  | (0.72) | (0.01) | (0.01) |
| Hybrid rice seed | 0.38 | 0.01 | 0.01 |
|  | (3.15) | (0.02) | (0.03) |
| Constant | 134.12*** | 4.93*** | 4.93*** |
|  | (15.42) | (0.15) | (0.13) |
|  |  |  |  |
| Observations | 1,396 | 1,396 | 1,396 |
| R-squared | -0.31 | -0.13 | -0.13 |

Robust standard errors in parentheses *** p<0.01, ** p<0.05, * p<0.1
