# Supplementary material for: Impact of training and digital extension services on agricultural technology adoption and rice yields
Source: PLoS One. 2025 Dec 5;20(12):e0337456. doi: 10.1371/journal.pone.0337456 (PMC12680215; doi:10.1371/journal.pone.0337456)
Supplement: S3 Table — (DOCX) [file pone.0337456.s003.docx]

S3 Table. Impact of agricultural technology adoption on rice yield (endogenous variable for the first stage: 2 doses of urea: 21 & 50 days)

|  | (2) | (3) | (4) |
| --- | --- | --- | --- |
|  | IVreg2 | IVreg2 | IVreg3 |
| VARIABLES | Total rice production(kg) | ln_rice_yield | ln_rice_yield |
|  |  |  |  |
| 2 doses of urea: 21 & 50 days | 40.75*** | 0.30** | 0.30** |
|  | (15.80) | (0.12) | (0.13) |
| Female respondent | -5.41* | -0.04 | -0.04 |
|  | (2.94) | (0.03) | (0.03) |
| Respondent's age | 0.07 | -0.00 | -0.00 |
|  | (0.17) | (0.00) | (0.00) |
| Hill dalit | -1.48 | -0.02 | -0.02 |
|  | (6.46) | (0.05) | (0.05) |
| Madheshi | 2.96 | 0.02 | 0.02 |
|  | (5.60) | (0.04) | (0.06) |
| Hill Janajati | 1.90 | -0.05 | -0.05 |
|  | (5.48) | (0.04) | (0.04) |
| Terai Janajati | -4.71 | -0.04 | -0.04 |
|  | (4.03) | (0.03) | (0.03) |
| Number of household members | -0.42** | -0.00** | -0.00* |
|  | (0.18) | (0.00) | (0.00) |
| Farm experience (years) | -0.08 | 0.00 | 0.00 |
|  | (0.14) | (0.00) | (0.00) |
| Female land ownership | -3.23 | -0.06 | -0.06** |
|  | (3.08) | (0.04) | (0.03) |
| Respondent's years of schooling | 0.16 | 0.00 | 0.00 |
|  | (0.29) | (0.00) | (0.00) |
| Share of land for rice cultivation | -0.97*** | -0.01** | -0.01*** |
|  | (0.31) | (0.00) | (0.00) |
| Canal irrigation | 6.51* | 0.05* | 0.05* |
|  | (3.53) | (0.03) | (0.03) |
| Canal and deep tubewell irrigation | 9.25** | 0.09*** | 0.09** |
|  | (3.90) | (0.03) | (0.03) |
| Number of land parcels | -0.64 | -0.00 | -0.00 |
|  | (0.60) | (0.00) | (0.01) |
| Less fertile land | -7.12*** | -0.04* | -0.04* |
|  | (2.76) | (0.02) | (0.02) |
| Use of mini-tiller | 2.03 | 0.02 | 0.02 |
|  | (2.96) | (0.02) | (0.03) |
| Use of thresher | -2.93 | -0.05 | -0.05 |
|  | (8.42) | (0.06) | (0.07) |
| Enough fertilizer available | -0.86 | -0.00 | -0.00 |
|  | (4.05) | (0.04) | (0.03) |
| Distance between household & cooperative | 1.68 | 0.02 | 0.02 |
|  | (2.00) | (0.02) | (0.02) |
| Distant to input market | -1.37** | -0.01 | -0.01 |
|  | (0.63) | (0.00) | (0.01) |
| Hybrid rice seed | 2.89 | 0.03 | 0.03 |
|  | (3.28) | (0.03) | (0.02) |
| Constant | 135.02*** | 4.92*** | 4.92*** |
|  | (15.94) | (0.15) | (0.12) |
|  |  |  |  |
| Observations | 1,396 | 1,396 | 1,396 |
| R-squared | 0.02 | 0.01 | 0.01 |

Robust standard errors in parentheses *** p<0.01, ** p<0.05, * p<0.1
