# Supplementary material for: Impact of training and digital extension services on agricultural technology adoption and rice yields
Source: PLoS One. 2025 Dec 5;20(12):e0337456. doi: 10.1371/journal.pone.0337456 (PMC12680215; doi:10.1371/journal.pone.0337456)
Supplement: S4 Table — (DOCX) [file pone.0337456.s004.docx]

S4 Table. Impact of agricultural technology adoption on rice yield (endogenous variable for the first stage: 2 doses of urea: land prep & 21 days)

|  | (2) | (3) | (4) |
| --- | --- | --- | --- |
|  | IVreg2 | IVreg2 | IVreg3 |
| VARIABLES | Total rice production(kg) | ln_rice_yield | ln_rice_yield |
|  |  |  |  |
| 2 doses of urea: land prep & 21 days | -91.23** | -0.53* | -0.53** |
|  | (37.85) | (0.30) | (0.26) |
| Female respondent | -8.39** | -0.06** | -0.06** |
|  | (3.77) | (0.03) | (0.03) |
| Respondent's age | -0.03 | -0.00 | -0.00 |
|  | (0.18) | (0.00) | (0.00) |
| Hill dalit | -9.00 | -0.08 | -0.08 |
|  | (7.02) | (0.05) | (0.05) |
| Madheshi | 1.14 | 0.01 | 0.01 |
|  | (7.60) | (0.05) | (0.06) |
| Hill Janajati | 4.31 | -0.05 | -0.05 |
|  | (7.27) | (0.05) | (0.04) |
| Terai Janajati | -3.94 | -0.04 | -0.04 |
|  | (4.76) | (0.04) | (0.03) |
| Number of household members | -0.74*** | -0.01*** | -0.01** |
|  | (0.24) | (0.00) | (0.00) |
| Farm experience (years) | -0.14 | 0.00 | 0.00 |
|  | (0.16) | (0.00) | (0.00) |
| Female land ownership | -3.93 | -0.06 | -0.06* |
|  | (3.95) | (0.04) | (0.03) |
| Respondent's years of schooling | 0.13 | 0.00 | 0.00 |
|  | (0.35) | (0.00) | (0.00) |
| Share of land for rice cultivation | -1.06** | -0.01** | -0.01*** |
|  | (0.45) | (0.00) | (0.00) |
| Canal irrigation | 13.59** | 0.09** | 0.09** |
|  | (5.41) | (0.04) | (0.04) |
| Canal and deep tubewell irrigation | 16.94** | 0.13** | 0.13*** |
|  | (7.16) | (0.06) | (0.05) |
| Number of land parcels | -0.08 | 0.00 | 0.00 |
|  | (0.79) | (0.01) | (0.01) |
| Less fertile land | -6.27* | -0.04 | -0.04 |
|  | (3.63) | (0.02) | (0.03) |
| Use of mini-tiller | 4.50 | 0.03 | 0.03 |
|  | (4.19) | (0.03) | (0.03) |
| Use of thresher | -9.03 | -0.08 | -0.08 |
|  | (10.35) | (0.07) | (0.08) |
| Enough fertilizer available | -3.51 | -0.01 | -0.01 |
|  | (5.36) | (0.04) | (0.04) |
| Distance between household & cooperative | -0.45 | 0.00 | 0.00 |
|  | (2.18) | (0.02) | (0.02) |
| Distant to input market | -0.56 | -0.00 | -0.00 |
|  | (0.77) | (0.01) | (0.01) |
| Hybrid rice seed | 3.20 | 0.03 | 0.03 |
|  | (3.98) | (0.03) | (0.03) |
| Constant | 177.35*** | 5.19*** | 5.19*** |
|  | (19.69) | (0.13) | (0.15) |
|  |  |  |  |
| Observations | 1,396 | 1,396 | 1,396 |
| R-squared | -0.49 | -0.16 | -0.16 |

Robust standard errors in parentheses *** p<0.01, ** p<0.05, * p<0.1
